# Sensor Integrated Load-Bearing Structures: Measuring Axis Extension with DIC-Based Transducers

**DOI:** 10.3390/s21124104

**Published:** 2021-06-15

**Authors:** Nassr Al-Baradoni, Peter Groche

**Affiliations:** Institute for Production Engineering and Forming Machines, Technical University of Darmstadt, 64287 Darmstadt, Germany; groche@ptu.tu-darmstadt.de

**Keywords:** sensor embedded structures, digital image correlation, multi-axis force/torque sensor, smart structures

## Abstract

In this paper we present a novel, cost-effective camera-based multi-axis force/torque sensor concept for integration into metallic load-bearing structures. A two-part pattern consisting of a directly incident and mirrored light beam is projected onto the imaging sensor surface. This allows the capturing of 3D displacements, occurring due to structure deformation under load in a single image. The displacement of defined features in size and position can be accurately analyzed and determined through digital image correlation (DIC). Validation on a prototype shows good accuracy of the measurement and a unique identification of all in- and out-of-plane displacement components under multiaxial load. Measurements show a maximum deviation related to the maximum measured values between 2.5% and 4.8% for uniaxial loads ($F_{x},\;F_{y},F_{z},M_{z})$ and between 2.5% and 10.43% for combined bending, torsion and axial load. In the course of the investigations, the measurement inaccuracy was partly attributed to the joint used between the sensor parts and the structure as well as to eccentric load.

## 1. Introduction

Force and torque measurements are applied in various fields, ranging from medical to industrial applications and aerospace. As digitization continues, application areas have continued to expand greatly, increasing the need for low-cost, structure-integrated and miniaturization-capable transducers. In the state of the art, traditional strain gauge-based sensors dominate the field of multi-axis force/torque sensors. In such measuring techniques, key sensor properties, such as sensitivity, sensor accuracy, measuring range and crosstalk between the measuring axes, depend significantly on the deformation behavior of the spring element and on the sensitive elements [1]. In order to ensure an optimal sensor performance, many studies introduce different designs for the spring element [2,3,4] as well as the application of alternative sensing elements like fiber grating [1,5] or semiconductor strain gauge [6,7]. Despite a large number of multi-axis force/torque sensors with different sensory characteristics in the current state of the art, their high cost prevents rapid implementation in many applications. According to Lee et.al, a tendency appears in the field of robotics to avoid the use of multi axial force/torque sensors, if their absence does not lead to a serious degradation of performance [8]. Considering the increasing research on multifunctional sensory structures, e.g., sensor-integrated metallic load-bearing structures such as in [9,10], the high cost and space requirements for the whole measuring chain seems to hinder the research in the integration of multi-axis force/torque sensors into such structures.

On the one hand, research efforts are being conducted to miniaturize and reduce the cost of strain gauge-based transducers through new design approaches, e.g., the three-axis force/torque sensor with only four strain gauges and a switchable quarter-bridge [11]. On the other hand, new sensors based on alternative measuring concepts, such as capacitive [12] or optical transducers, are being investigated primarily with the aim of providing a cost-effective and miniaturizable alternative to conventional strain gauge-based transducers. In particular, optical-based non-contact sensors have gained special attention. They stand out due to low-cost, simple design and encourage structural integration by eliminating the spring element as well as miniaturization. In the field of optical force/torque measurement, Tar et al. presented a 3D tactile force sensor in which light emitted by LEDs is received by deformable hollow mirror rubber hemispheres and reflected into light-emitting diodes. The magnitude and direction of the grip force is then determined by the change in intensity at the light diodes. This enables force sensing in the compliant gripper of a robot with a stable grip [13]. A similar principle of operation is presented by Noh et al. in the form of a three-axis force/torque sensor for easy installation in the structure of a manipulator arm [14]. Al-Mai et al. presented firstly a 3-axis, and then later a 6-axis fiber optic force/torque sensor in which the generated and reflected light is propagated with high accuracy and low noise through a pair of optical fibers from the LED to the mirrors and then back to the photodiode (LTV) [15,16]. Xie et al. adopted the optical measurement concept due to the requirement of application in a magnetic resonance imaging (MRI) environment where no metallic component could be used. Unlike the previous measurement concepts, where the change in light intensity and thus the displacement due to loading was measured by evaluating the proportional voltage value of the applied photodiodes, here the intensity change was pixel-based and measured with a camera [17]. Díez et al. presented an optical force sensor based on a different measurement concept, where the load on the structure causes the displacement of a built-in converging lens. The light distortion caused during the lens displacement is measured by a 2 × 2 photo-detector matrix and interpreted as a bending proportional signal [18].

All these presented measurement concepts are based on the direct evaluation of the intensity change as a proportional quantity to the load condition. Despite the mainly economic advantages of the presented optical sensors in terms of more flexible and lighter designs, a high softness of the structures is still required to produce measurable displacements. Hence, optical sensors have been used for force measurements in more compliant structures such as plastic and rubber. Their measuring resolution in terms of the required deformation is quite far behind the achievable performance of conventional strain gauge-based or piezoelectric sensors. According to Berkovic et al., an optical sensing with higher resolution, e.g., in the range of 1 µm, requires relatively expensive and complex measuring technologies, such as interferometry or confocal sensing techniques [19].

In the field of stress analysis in solid mechanics, on the other hand, high-resolution optical techniques such as holographic interferometry, speckle interferometry and Moiré interferometry as well as non-interferometric techniques, such as the grating method [20,21] and digital image correlation (DIC), are well-established [22].

Digital image correlation refers to a contactless measurement technique that acquires images of an object, stores them digitally, and conducts image analysis to extract whole-field shape, deformation, motion measurements, or in combination [23]. With its clear advantages in comparison to other high resolution measuring techniques, which include simple experimental setup, easy implementation, high robustness to environmental vibrations and light fluctuations, and wide applicability due to adjustable temporal and spatial resolutions, the DIC technique has become a powerful and flexible tool for shape, motion and deformation measurement for different materials and structures, on different temporal and spatial scales, and in different experimental environments [24]. Applications therefore range from very large, such as bridges [25], to the nano-meter scale in precision mechanics instrumentation [26,27]. In addition to typical applications for the computation of strain or displacement, the DIC technique is also used for force measurement, such as the detection of cutting forces [28] in a process or for monitoring the clamping forces of screws during assembly [29].

### Fundamentals of DIC

In the classical applications of DIC, the surface of an object of interest is prepared by applying a random speckle pattern. The pattern serves as an information carrier and is expected to have unique, non-periodic and stable greyscale features. For this purpose, different methods like spraying, spin coating and lithography exist [24]. To avoid aliasing, a speckle size of maximum 3 × 3 pixels is desired [30]. Subsequently, the coordinates of the subset with a predefined area in the reference image are selected and then sought in the target image within a predefined area there. The correlation values for the whole search field are stored in a correlation matrix, and the coordinates of the highest correlation value are assigned to the new position of the searched subset center. Small subsets increase the risk of misclassification because the uniqueness decreases. However, for small subsets, the local strain excesses can be measured with higher resolution [31]. Especially the size of the subset and the search field have a strong impact on the computation time [32]. Similar to the correlation methods, different algorithms can be used to increase the resolution by so-called subpixel registration. Yang et al. achieved measurement resolution as low as 0.01 pixels to increase the measurement resolution in dynamic testing of microelectromechanical systems (MEMS) [33]. Applying optical magnification can significantly increase the measurement resolution. In [34], a shift of the micromechanical device with a measurement accuracy within the picometer range could be achieved.

Two-dimensional displacements or deformations in the plane are measured with the simple installation of a single camera DIC [22]. In this case the examined surface is 90° with respect to the optical axis of the camera. A three-dimensional acquisition of the deformations, on the other hand, is carried out with so-called stereo-DIC or 3D-DIC methods. This is usually achieved by synchronized image acquisition from two cameras, each rotated by 45° to the image plane [35]. Using optical elements such as prisms, mirrors or diffraction gratings and the fundamental laws of optics, numerous techniques for single-camera stereo DIC have emerged in research, enabling three-dimensional shape and deformation measurements [36]. Although DIC is currently one of the most popular and active methods in the field of experimental mechanics with the lowest requirements for an operating environment, its measurement accuracy as a non-interferometric method strongly depends on the illumination conditions during the measurement. Variations in the illumination conditions and thus in the quality of the acquired images directly affect the measurement quality [37]. Depending on the desired measurement resolution, very strong illumination is usually required to obtain high resolution.

So far, the applications of DIC have mostly been limited to fixed measurement systems and the acquisition of displacement fields. However, in the area of sensor-integrated metallic load-bearing structures and machine elements, the highly adjustable measurement resolution, the low costs and robust components of the DIC seem to be very attractive. The high pixel counts of standard image sensors in the smallest possible space, combined with a suitable sensor design, are particularly promising for the detection of multi-axial loads. In addition, the cavities in the structures provided for sensor integration offer laboratory-like lighting conditions, which increases measurement accuracy.

In this paper we investigate the utilization of a basic optical DIC-based technique as a structure-integrated multi-axis force/torque sensor. The aim is to achieve a cost-effective extension of the measuring axes, with regard to the entire conventional measurement chain, and thus to form integrated force and torque transducers without having to integrate a complex designed spring element. First, the technique is modified for use as a structurally integrated multi-axis force/torque sensor. The achievable sensory properties, like measuring accuracy and linearity, will then be investigated using a prototype on a test bench. Special emphasis is placed on accurate load classification when multiple loads are applied to the structure. For the achieved measurement accuracy, the robustness of the measurement concept in particular has to be estimated, and an orientation for the integrability of optical high-resolution measurement concepts in metallic load-bearing structures as well as the requirements of the joining process have to be determined. Firstly, an overview of the design of sensory structures and machine elements is provided, followed by a description of the design of the DIC-based multi-axis force/torque sensor. Subsequently, experiments based on a prototype and the achieved results are presented. Finally, a summary of the achieved results discusses the applicability of camera-based measuring concepts for the production of multi-axis force/torque sensory load-bearing structures, along with its limitations and special requirements.

## 2. Methods

### 2.1. Background of Sensor Integrated Load-Bearing Structures

**Figure 1:**
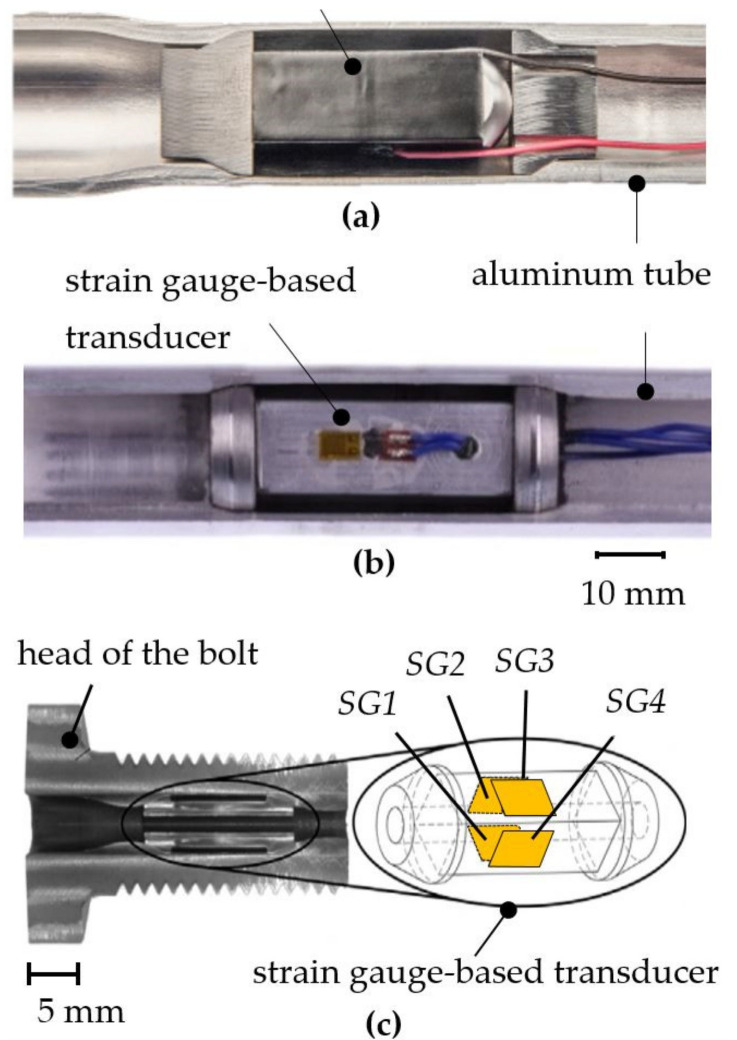
Load-bearing structures with integrated strain gauge-based and piezo electric transducers produced by rotary swaging. (**a**,**b**) Sensory hollow shafts [45]. (**c**) Sensory bolt [46].

In the context of advancing digitalization, the demand for additional sensory or adaptive functions is rising for conventional load-bearing structures and machine elements. For widespread implementation, their functionality must be cost-effectively expanded while fully maintaining the mechanical functionality. To meet this requirement, various approaches are being developed for manufacturing multifunctional structures in which sensors or actuators are integrated into metallic structures. For example, piezoelectric transducers have been integrated into metallic structures by die casting [38,39] or through additive manufacturing [40,41]. In this field, joining by forming has proven to be a particularly suitable and economic technology [42,43,44]. Here, transducers are pretensioned and integrated into hollow structures through force- or force- and form-fit joining by means of forming techniques, like rotary swaging (see Figure 1). Despite the successful manufacturing of sensory load-bearing structures and machine elements whilst maintaining conventional mechanical functionality, their force/torque measuring capability is limited to one or two measuring axes. With regard to the joining process, presented in [43], the integration of an electro-mechanical transducer for multi-axis force/torque measuring requires a new complex process design to ensure an anisotropic deformation behavior of the transducer. This requires not only the creation of the force/form-fit connections between the transducer and the structure in all measuring directions, but also the alignment of a specific stress state on the transducer after joining. However, this would be complex due to the nature of the forming process used.

### 2.2. The DIC-Based Force/Torque Sensor

#### 2.2.1. Sensor Design

**Figure 2:**
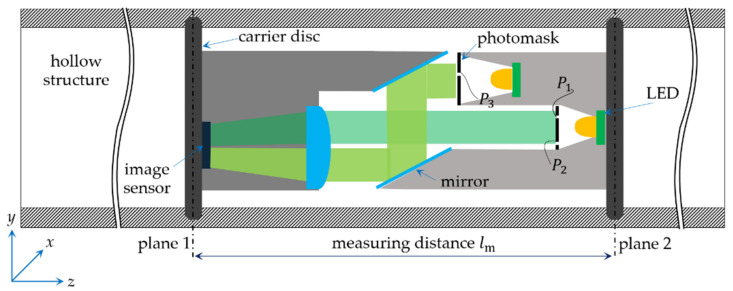
Principle of function for multi-axis force/torque measurement.

The basis of the measurement concept is to determine the three-dimensional displacement of a structure under load by measuring the resulting displacement between two planes on the structure. Essentially, the sensor design comprises a camera and an object mounted on two carrier disks. These carrier disks are parallel and fixed in the structure at two spatial points with distance $l_{m}$, as shown in Figure 2. Relative displacements occurring between the two planes are captured by the camera images and measured by means of DIC.

While in-plane displacements (*x–y*) of the two carrier disks (caused by bending or torsional loads) are simply acquired due to the parallel arrangement of the camera to the object, out-of-plane displacements (subject to axial load, *z*-axes) require an appropriate optical extension of the system. A special set-up with two mirrors translates out-of-plane displacements into in-plane displacements, meeting the requirements for a very simple and robust setup in limited space. The design resembles a periscope, with two mirrors tilted at 45°, which change their relative positions as the distance between the two carrier disks changes under axial load, as shown in Figure 2. The area of the image sensor is divided into two parts. One part captures the direct beam with the information about in-plane displacements and the other part captures the mirrored beam with information about displacements in the third dimension. The mirrored object part is placed closer to the camera in order to keep the light path between both parts of the image as equal as possible. To uniquely assign the in-plane displacements caused by bending or twisting loads, the evaluation of the motion of two points ($P_{1}$ and $P_{2}$) on the object is required. Out-of-plane displacements can be traced by displacement evaluation at only one point, $P_{3}$, on the mirrored ray, as indicated in Figure 2.

To achieve a high degree of design flexibility with increased DIC matching accuracy at minimized computational effort, the pattern is realized as a back-illuminated foil photomask with transparent points, with predefined positions and diameter (as also shown in [47]). Generally, the smaller the features (speckles) in a pattern, the less computational effort is required for correlation and the higher the measuring resolution is. The diameter of the points, however, must cover a maximum of three pixels of the image sensor used to avoid aliasing [30]. To avoid any mismatch during the correlation and thus to maximize the measuring sensitivity, the positions of the points can be chosen considering the highest possible displacements.

#### 2.2.2. Determination of the System Behavior

If the structure is loaded with only one bending load, $F_{\mathrm{xy}}$, the amplitude and direction of this resulting deflection ${\vec{\delta }}_{\mathrm{xy}}$ can be determined by the displacements $\Delta P_{1}$ and $\Delta P_{2}$, which are equal in magnitude and direction. For a torsional load $M_{t}$, the angle of twist $\varphi \sim M_{t}$ is calculated from the unequal displacement as the angle between the two vectors, connecting $P_{1}$ and $P_{2}$ before and after the displacement, as follows:

(1) $${\vec{v}}_{1}=\left(\begin{array}{c} P_{1\_}x-P_{2\_}x\\ P_{1\_}y-P_{2\_}y \end{array}\right),{\vec{v}}_{2}=\left(\begin{array}{c} (P_{1\_}x+\Delta P_{1\_}x)-\left(P_{2\_}x+\Delta P_{2\_}x\right)\\ (P_{1\_}y+\Delta P_{1\_}y)-\left(P_{2\_}y+\Delta P_{2\_}y\right) \end{array}\right)$$

where $P_{i\_}x$ and $P_{i\_}y$ are the x- and y-components of the initial positions of $P_{1}$ and $P_{2}$ and $\Delta P_{i\_}x$ and $\Delta P_{i\_}y$ are the x- and y-components of each displacement, respectively.

The angle of twist $\varphi \sim M_{t}$ is then given by the dot and cross product of two vectors as follows:

(2) $$\varphi =\arccos\left(\frac{{\vec{v}}_{1}\cdot {\vec{v}}_{2}}{\left|{\vec{v}}_{1}\right|\times \left|{\vec{v}}_{2}\right|}\right)$$

Similarly, a mere compression or elongation of the structure due to an axial load $F_{z}$ results in a change of the measuring span $l_{m}\pm \Delta l_{m}$, which is translated into the radial displacement $\Delta P_{3}$ in the image plane.

In case of multi-axis loads, the translational (deflection, ${\vec{\delta }}_{\mathrm{xy}}$) and rotatory (angle of twist, φ) components of in-plane displacements at “direct” image segments are clearly determined by a known angle of twist φ and center of rotation ${\vec{c}}_{\mathrm{CR}\_d}$ as follows:

(3) $${\vec{\delta }}_{\mathrm{xy},i}={\vec{c}}_{i}+\Delta {\vec{P}}_{i}-{\vec{c}}_{\mathrm{CR}\_d}-\left(\begin{array}{cc} \cos\left(\varphi \right) & -\sin\left(\varphi \right)\\ \sin\left(\varphi \right) & \cos\left(\varphi \right) \end{array}\right)\left({\vec{c}}_{i}-{\vec{c}}_{\mathrm{CR}\_d}\right),\;i=1,2$$

where $\Delta {\vec{P}}_{i}$ is the displacement detected by DIC and ${\vec{c}}_{i}$ is the corresponding initial position of the evaluation point $P_{i}$. The deflections ${\vec{\delta }}_{\mathrm{xy},1}$ and ${\vec{\delta }}_{\mathrm{xy},2}$ are subsequently averaged to ${\vec{\delta }}_{\mathrm{xy}}$ and then separated into $\delta_{x}$ and $\delta_{y}$. Alternatively, the translational displacement of the center of rotation can be achieved by applying the barycentric coordinates. For this purpose, three evaluation points are required, to create a triangle enclosing the center of rotation.

**Figure 3:**
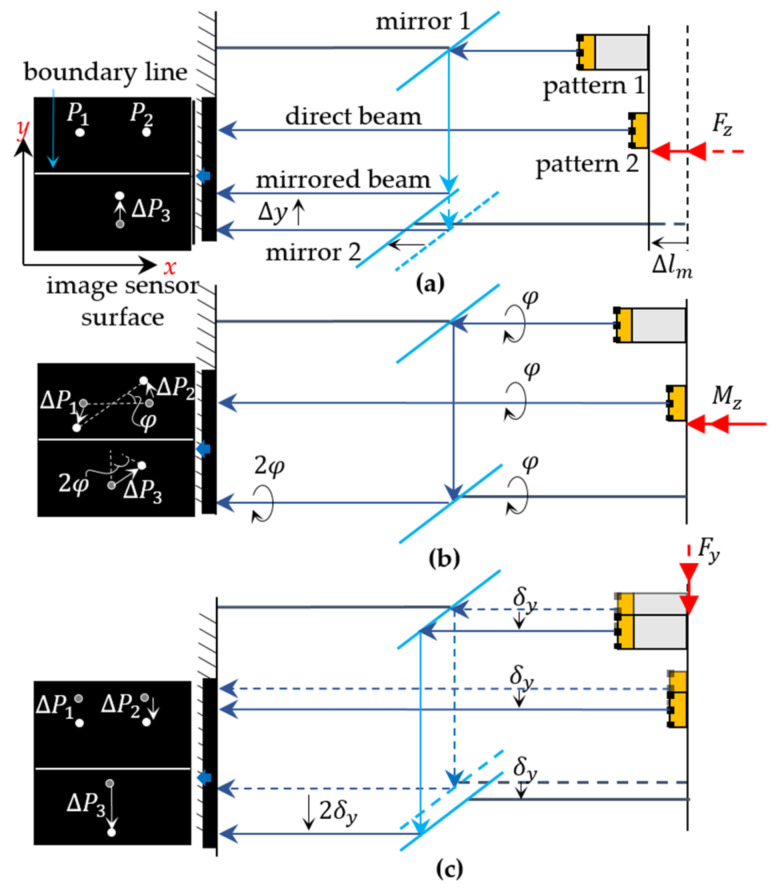
Visualization of the crosstalk behavior in the mirrored image segment by in-plane displacements. (**a**) compression (**b**) torsion (**c**) deflection.

Due to the comparatively small compression or elongation of load-bearing structures, the effect on in-plane displacement due to change in feature size ($\frac{\Delta A}{A}\approx \frac{\Delta l_{m}}{l_{m}})$, is negligible. A critical disappearance of the feature upon elongation of the structure can be considered when designing the feature size. In contrast, all in-plane displacements cause crosstalk on out-of-plane displacements appearing at the “mirrored” image segment. In this case, the displacement between pattern 1 to mirror 1 and the displacement between the mirrors to each other determine the resulting displacement point $\Delta P_{3}$ on the mirrored image segment. Figure 3 shows the displacements induced in both direct and mirrored image segments under load with axial force $F_{z}$, torque $M_{z}$ and bending force $F_{y}$, which is perpendicular to the theoretical “boundary line” between the direct and mirrored rays.

**Figure 4:**
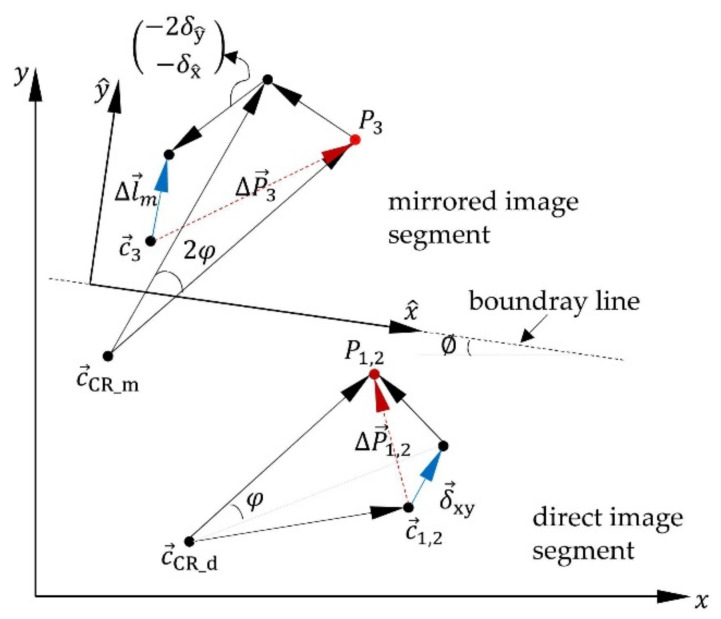
Crosstalk behavior in both in- and out-of-plane displacement and the possibility of determining the causing loads.

While compression or elongation of the structure causes a displacement between the mirrors, resulting only in displacements $\Delta P_{3}=\Delta y$ on the mirrored image segment (Figure 3a), the angle of twist φ appears doubled in the mirrored image because of the displacements between pattern 1 and mirror 1 and between mirror 1 and mirror 2 (Figure 3b). These two displacement types (between pattern 1 and mirror 1 and between the two mirrors) cause, in case of structure deflection under bending force $F_{\mathrm{xy}}$, doubled displacements on the mirrored image segment $\left(\Delta P_{3}=2\Delta P_{1,2}\right)$, if the induced deflection is perpendicular to the boundary line (Figure 3c). They cause a simple displacement if the induced deflection is along the boundary line. The illustrated displacement at the mirrored image segment $2\delta_{y}$ caused by $F_{y}$ in Figure 3c is considered as an ideal scenario, by having the boundary line, whose orientation on the image sensor’s surface is defined by the rotation of the mirrors around the z-axis, is perfectly parallel to the x-axis of the image sensor. However, this cannot be ensured due to the deviations to be expected during sensor installation. For this reason, it will be further considered that the boundary line is rotated at an angle ∅ to the x-axis of the image sensor. Therefore, the “boundary line coordinate system” $\hat{x}-\hat{y}$ is used, which is rotated at an angle ∅ to the image sensor coordinate system x−y. In this way, the perpendicular and parallel displacement components to the boundary line can be determined as a function of the angle ∅, as illustrated in Figure 4. 

After calculating both in-plane translational and rotatory displacements, the strain of the structure caused by axial load $\Delta \vec{l_{m}}$ can be determined with a known center of rotation of the mirrored image segment ${\vec{c}}_{\mathrm{CR}\_m}$ as follows:

(4) $$\Delta \vec{l_{m}}=R\left(\emptyset \right)\cdot \left({\vec{c}}_{3}+\Delta {\vec{P}}_{3}-{\vec{c}}_{\mathrm{CR}{\_}_{m}}-\left(\begin{array}{cc} \cos\left(2\varphi \right) & -\sin\left(2\varphi \right)\\ \sin\left(2\varphi \right) & \cos\left(2\varphi \right) \end{array}\right)\cdot \left({\vec{c}}_{3}-{\vec{c}}_{\mathrm{CR}\_m}\right)\right)\;-\left(\begin{array}{cc} 1 & 2 \end{array}\right)\cdot R\left(\emptyset \right)\cdot {\vec{\delta }}_{\mathrm{xy}}$$

with

(5) $$R\left(\emptyset \right)=\left(\begin{array}{cc} \cos\left(\emptyset \right) & \sin\left(\emptyset \right)\\ -\sin\left(\emptyset \right) & cos\left(\emptyset \right) \end{array}\right)$$

where $\Delta {\vec{P}}_{3}$ is the displacement detected by DIC and ${\vec{c}}_{3}$ is the initial position of the evaluation point $P_{3}$ on the mirrored image segment.

The effect of the rotatory crosstalk with the angle 2φ is first eliminated from the displacement $\Delta {\vec{P}}_{3}$ by coordinate transformation with 2φ. Furthermore, a second coordinate transformation is performed into the boundary line coordinate system with the angle ∅ of the intermediate result together with the translational in-plane displacements ${\vec{\delta }}_{\mathrm{xy}}$, doubling the $\hat{y}$-component. The angle ∅ can be determined by means of a strictly uniaxial bending load ($F_{x}$ or $F_{y}$), which leads to a one-dimensional displacement at the direct image segment ($\Delta P_{1}$ and $\Delta P_{2}$) and a two-dimensional displacement on the mirrored image segment ($\Delta P_{3}$). The angle ∅ can then be calculated as:

(6) $$\emptyset =\arctan\left(\frac{\Delta P_{3}\_y}{\Delta P_{3}\_x}\right)$$

where $\Delta P_{3}\_x$ and $\Delta P_{3}\_y$ are the x- and y-components of the calculated displacement $\Delta P_{3}$.

Figure 4 illustrates the driven correlation in Equations (3) and (4). The correlations to compensate the crosstalk of in-plane displacements (${\vec{\delta }}_{\mathrm{xy}}$ and φ) from the out-of-plane displacement $\Delta P_{3}$ assume, however, a parallel alignment of the mirrors to each other. In practice, some inaccuracy, depending on the accuracy of the assembly process, is to be expected. Furthermore, inaccurate deformation behavior of the structure, due to various sources of uncertainty in manufacturing and assembling [48], is another possible source of inaccurate crosstalk compensation. Consequently, an investigation of the crosstalk behavior is essential as part of a calibration process.

Once the calibration values have been determined, the applied forces and torques can be given as follows:

(7) $${\left(F_{x}\;F_{y}\;F_{z}\;M_{z}\right)}^{T}=\vec{kv}\times \left(\delta_{x}\;\delta_{y}\;\Delta l_{m}\;\varphi \right)$$

where $\vec{kv}$ represents the determined calibration values.

Assuming that the bending forces $F_{x}$ and $F_{y}$ act only outside the measuring zone and there is a homogenous structural deflection, the bending moments can be calculated by:

$$M_{x}=F_{x}l_{m}\;\mathrm{and}\;M_{y}=F_{y}l_{m}$$

## 3. Evaluation and Results

### 3.1. Design of the Prototype

**Figure 5:**
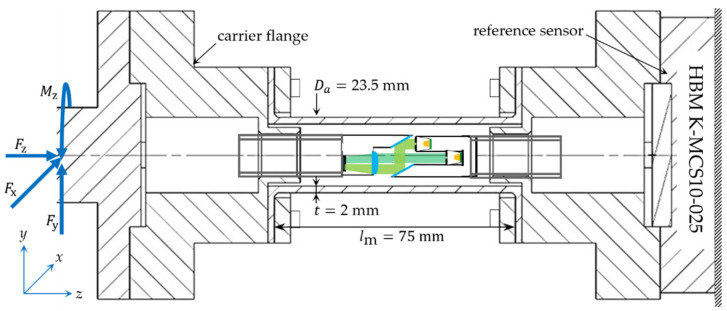
Design of the prototype used to evaluate the functional principal.

To evaluate the measurement concept, a prototype was designed, in which the displacement occurring during the loading of a hollow tube is measured in two parallel planes. The prototype must ensure parallel fixing of the two sensor parts to the structure. Furthermore, it is essential to exclude any deformations in the joining area between the sensor parts and the structure, which can lead to a falsification of the determined displacement. Therefore, in the designed prototype, the pattern and the camera are attached to two rigid flanges (carrier flange) with one screw. The two flanges are then bolted to both ends of a tubular structure (steel, length = 75 mm, diameter = 23.5 mm and thickness = 2 mm), as shown in Figure 5.

**Figure 6:**
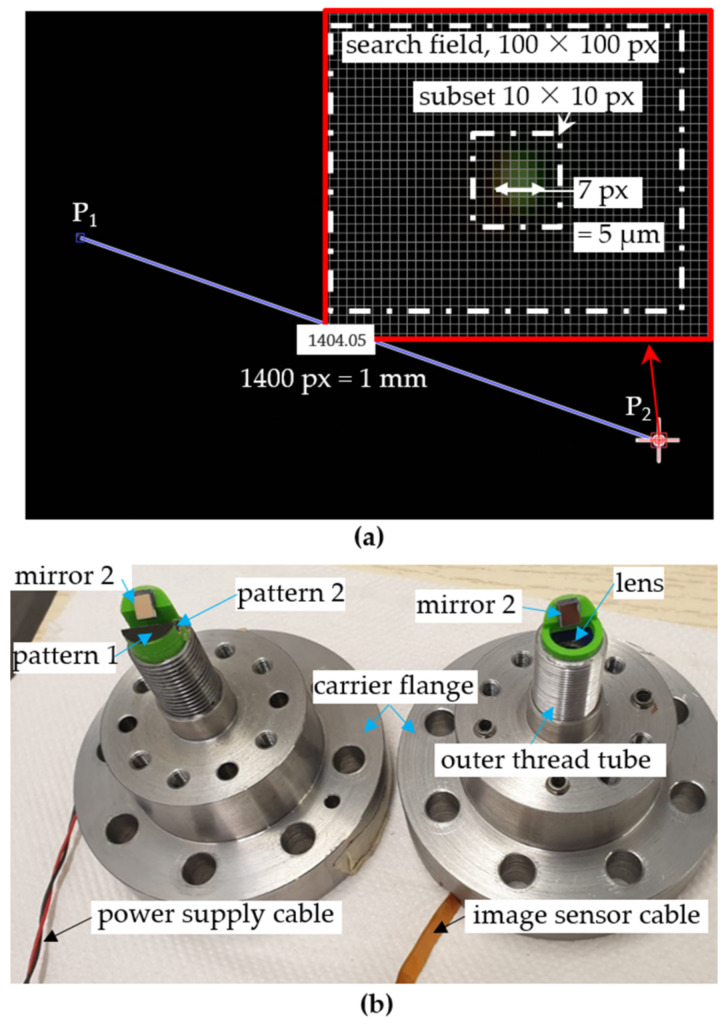
(**a**) Design features of the photomask for the direct image segment and adjusted DIC algorithm parameters, (**b**) carrier flanges with both senor parts.

The loads are then applied to the left side of the structure, causing deformation of the tubular structure. The applied loads are measured on both the DIC-based sensor and the multi-axis force/torque reference sensor (HBM: K-MCS10-025). To minimize optical aberrations and to sufficiently focus the points $P_{1-3}$ at the maximum expected elongation or compression of the structure (±0.0258 mm at $F_{z}$ = ±10 kN), the camera was designed to provide an optical magnification of m = −1 by using a dual-lens system. Simple optical verification in WinLins 3D Basic shows good imaging quality, with a depth of field of ±0.05 mm. The patterns are therefore imaged 1:1 by an active area on an image sensor (Omnivision OV5647) of 3673.6 μm × 2738.4 μm with a pixel size of 1.4 μm × 1.4 μm. Image acquisition was performed using a Raspberry Pi device. The used photomask is produced by JD PHOTO DATA and has a point diameter of 5 µm, see Figure 6a. Both the camera, the patterns and the mirrors were mounted in the tube with an external thread for distance adjustability using 3D printed parts, see Figure 6b.

To determine the center of rotation in the mirrored image segment ${\vec{c}}_{\mathrm{CR}\_m}$, another temporary evaluation point $P_{4}$ was placed in the photomask of pattern 1 outside the expected displacement field of the evaluation point $P_{3}$ (at a distance of 500 µm from $P_{3}$). The centers of rotation ${\vec{c}}_{\mathrm{CR}\_d}$ and ${\vec{c}}_{\mathrm{CR}\_m}$ are be determined by calculating the intersection points of two linear functions, which are described by the two points of each pattern before and after a rotation caused by a mere torsional load. The calculation of the displacement due to the DIC was performed in Matlab, where integer displacement (whole pixel displacement) was calculated using zero-normalized cross correlation after Giachetti et al. [49]. For sub-pixel displacement, we applied the cores-fine search method with linear interpolation, as described in Simončič et al. [50]. The center coordinates for the evaluation points $P_{1-3}$ were first determined by their high grayscale values. For DIC computing, a subset size of 10 pixels and a pixel shift of 100 pixels was specified, as shown in Figure 6. The displacements are computed with a sub-pixel registration of 0.125 pixels ≈ 0.09 µm. Following this, uniaxial loads ($F_{x}$, $F_{y}$,$\;F_{z}$, $M_{z}$) were manually applied within the defined loading range of the structure ($F_{x}$ and $F_{y}$ between ±250 N, $M_{z}$ between ±65 Nm and $F_{z}$ between ±3 kN). To evaluate the sensor performance independently from the stiffness of the host structure, the measured displacement components (φ, $\delta_{x}$, $\delta_{y}$ and Δ$l_{m}$) were then compared with the reference components resulting from the measured loads on the reference sensor and the theoretically resulting displacement in the tube structure. After that, the centers of rotation ${\vec{c}}_{\mathrm{CR}\_d}$ and ${\vec{c}}_{\mathrm{CR}\_m}$ were determined by uniaxial torsional loading. Furthermore, the crosstalk behavior between in-plane and out-of-plane displacements and the angle of boundary line ∅ were investigated. After complete characterization of the sensor behavior, multiaxial loads ($F_{x}$ + $F_{y}$ + $M_{z}$) and then three-dimensional multiaxial loads ($F_{x}$ + $F_{y}$ + $F_{z}$ + $M_{z}$) were applied and the resulting displacements in their components are calculated (φ, $\delta_{x}$, $\delta_{y}$, Δ$l_{m}$) according to Equations (1)–(6). All load components are increasing and decreasing together along the load ranges, respectively. The loads were applied in about 10 steps.

### 3.2. Results

Firstly, the results of uniaxial loads are shown. Afterwards, the crosstalk behavior between in-plane and out-of-plane displacement is investigated, the driven correlations in Figure 3 and Equation (4) are experimentally evaluated and both centers of rotation ${\vec{c}}_{\mathrm{CR}\_d}$ and ${\vec{c}}_{\mathrm{CR}\_m}$ are determined. Finally, the structure is loaded with in-plane displacement causing multiaxial loads ($F_{x}+F_{y}+M_{z}$) and then with the whole load components ($F_{x}+F_{y}+F_{z}+M_{z}$) and the results are shown. At the end of this subsection, the obtained regression models for each displacement component ($\delta_{x}$, $\delta_{y}$, φ, Δ$l_{m}$) and the achieved accuracy in each loading case are determined and compared, see Table 2. This is followed by a discussion of possible causes of the measurement deviation.

#### 3.2.1. Results of Uniaxial Loading

**Figure 7:**
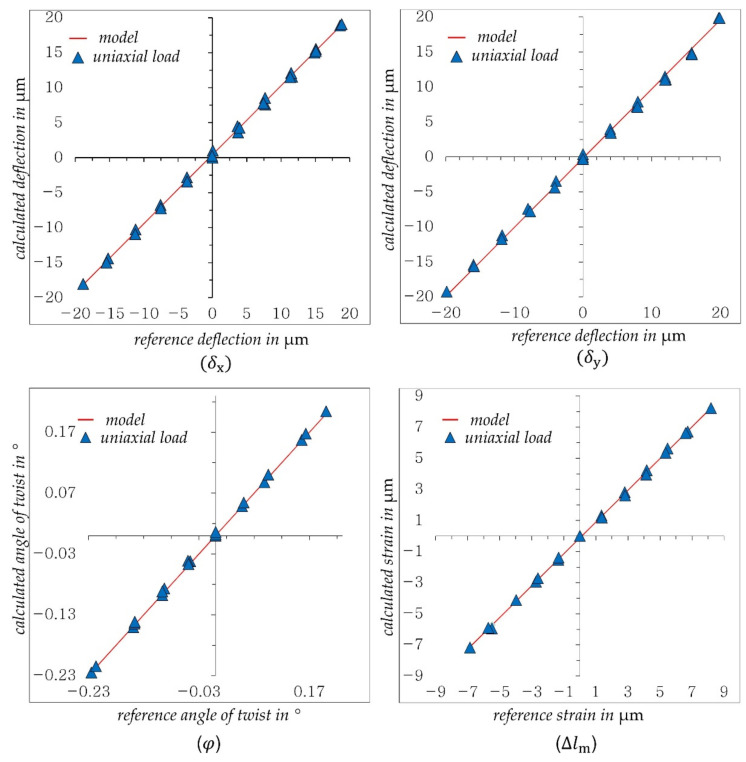
Measurement accuracy achieved for uniaxial in comparison with reference values.

Figure 7 presents the achieved results for uniaxial loads ($F_{x},F_{y},\;F_{z},M_{z}$).

As shown in Figure 7, the deflections $\delta_{x}$ and $\delta_{y}$, the twist angle φ and the strain Δ$l_{m}$ determined by DIC show very good and linear agreement with the reference values. The regression models shown here are used in the following tests.

#### 3.2.2. Determining the Crosstalk Behavior

**Figure 8:**
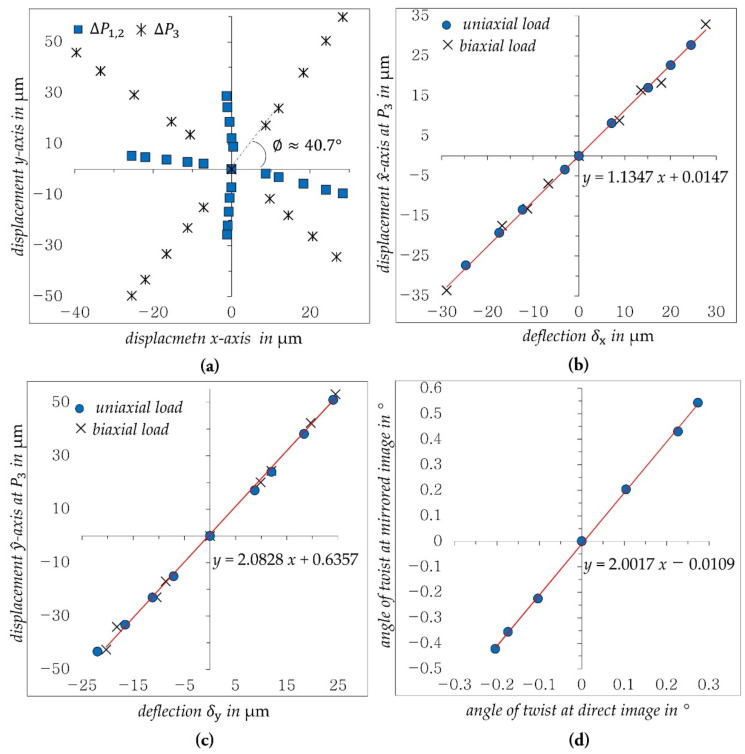
Characterization of sensor behavior, (**a**) determined angle of boundary line ∅, (**b**) determined crosstalk between $\delta_{x}$ and $P_{3}\_\hat{x}$ for uniaxial ($F_{x}$) and biaxial ($F_{\mathrm{xy}}$) loading, (**c**) determined crosstalk between $\delta_{y}$ and $P_{3}\_\hat{y}$ for uniaxial ($F_{y}$) and biaxial ($F_{\mathrm{xy}}$) loading, (**d**) crosstalk of the twist angle φ on mirrored image segment.

The angle ∅, by which the mirrored beam is twisted to the image sensor coordinate system, and the crosstalk between in-plane displacement components $\delta_{x}$ and $\delta_{y}$ and the out-of-plane displacement Δ$l_{m}$ are determined by uniaxial loading of the structure with $F_{x}$ and $F_{y}$, respectively; see Figure 8a. Thus, the angle ∅ can be calculated according to Equation (6). Afterwards, biaxial bending loads $F_{\mathrm{xy}}$ are applied, the resulting displacement of $\Delta P_{3}$ at the mirrored image segment is transformed into the boundary line coordinate system ($\hat{x}-\hat{y})$ and the correlations between the resulting in-plane displacement components ($\delta_{x}$ and $\delta_{y}$) and the displacement components $\Delta P_{3}\_\hat{x}$ and $\Delta P_{3}\_\hat{y}$ at the mirrored image segment after transformation the coordinate system with ∅ are determined; see Figure 8b,c. In case of a torsion load, the crosstalk was investigated by determining the resulting angle of twist of both image segments. Figure 8d shows the resulting correlation between the twist angle φ and the corresponding rotation of the mirrored image segment.

As shown in Figure 8b–d, the slopes of the determined regression models are only slightly different from the theoretical values in Figure 3. Equation (4) is therefore modified with the new slopes to:

(8) $$\Delta \vec{l_{m}}=R\left(\emptyset \right)\cdot \left({\vec{c}}_{3}+\Delta {\vec{P}}_{3}-{\vec{c}}_{\mathrm{CR}\_m}-\left(\begin{array}{cc} \cos\left(2\varphi \right) & -\sin\left(2\varphi \right)\\ \sin\left(2\varphi \right) & \cos\left(2\varphi \right) \end{array}\right)\cdot \left({\vec{c}}_{3}-{\vec{c}}_{\mathrm{CR}\_m}\right)\right)\;-\left(\begin{array}{cc} 1.135 & 2.081 \end{array}\right)\cdot R\left(\emptyset \right)\cdot {\vec{\delta }}_{\mathrm{xy}}$$

The inaccurate application of the displacement $\delta_{x}$ and $\delta_{y}$ as shown in Figure 8a is caused by the inaccurate manual load adjustment in the designed test bench. The calculated angle ∅, however, seems to be very accurate, as can be seen in the good correlation of the displacement $\Delta P_{3}$ along the $\hat{x}$- and $\hat{y}$-axis to the deflections $\delta_{x}$ and $\delta_{y}$, respectively, for both uniaxial und biaxial loading.

#### 3.2.3. Determining of Centers of Rotation

**Figure 9:**
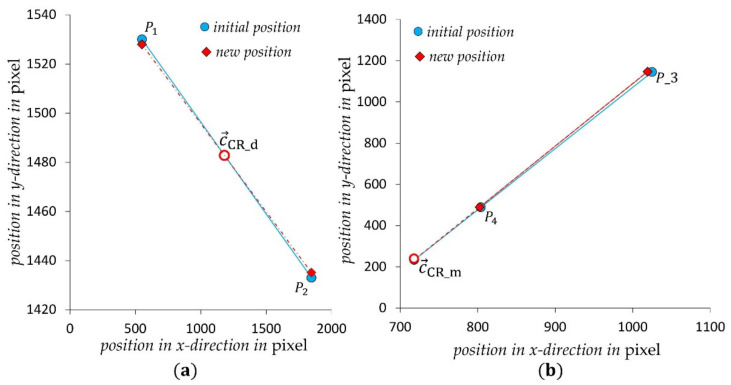
Positions of points $P_{1-4}$ before and after the torsional load and positions of the centers of rotation (**a**) for the direct image segment and (**b**) for the mirrored image segment.

As described in Section 3.1, the centers of rotation ${\vec{c}}_{\mathrm{CR}\_d}$ and ${\vec{c}}_{\mathrm{CR}\_m}$ can be determined by calculating the intersection points of two linear functions, which are described by the two points of each pattern before and after a rotation caused by a mere torsional load. As can be seen in Table 1, the initial positions (${\vec{c}}_{i}$) of points $P_{1-4}$ are given. After loading the structure with $M_{z}=40\;\mathrm{Nm}$, an image is acquired and the resulting displacements at each point ($\Delta {\vec{P}}_{i}$) are determined by means of the DIC algorithm. This results in the new positions (${\vec{c}}_{i}+\Delta {\vec{P}}_{i}$). The intersection points of two linear functions connecting two points $P_{1,2}$ and $P_{3,4}$ before and after the displacement, result in the corresponding centers of rotation for each image segment (${\vec{c}}_{\mathrm{CR}\_d}$,$\;{\vec{c}}_{\mathrm{CR}\_m}$). See Table 1 and Figure 9.

As visualized in Figure 9, the determined center of rotation for the direct image segment lies nearly in the middle of the image sensor surface and matches the position of the direct pattern to the image sensor in Figure 2. For the mirrored image segment, the center of rotation lies in the lower part of the image sensor, due to the offset in the height and the double mirroring of the mirrored pattern, see Figure 2 and Figure 3. The observed shift to the left side of the image sensor had to be caused by the rotation of the mirrors to the image to the image sensor by the determined angle ∅.

#### 3.2.4. Results of Multiaxial Loads ($F_{x}$ + $F_{y}$ + $M_{z}$)

With the known center of rotation for the direct image segment ${\vec{c}}_{\mathrm{CR}\_d}$, in-plane displacement causing the load combination $F_{x}+F_{y}+M_{z}$ can be applied. The measured displacements $\Delta P_{1}$ and $\Delta P_{2}$ are then calculated to $\delta_{x}$, $\delta_{y}$ and φ according to Equations (1)–(3). First, an exemplary calculation is performed to demonstrate, how to identify the translational ($\delta_{x}$ and $\delta_{y}$) and rotational (φ) displacement components from the resulting displacement of points $P_{1}$ and $P_{2}$ in a combined bending and torsion load ($F_{x}=-154.4\;N$, $F_{y}=-158.2\;N$ and $M_{z}=40\;\mathrm{Nm}$). 

**Figure 10:**
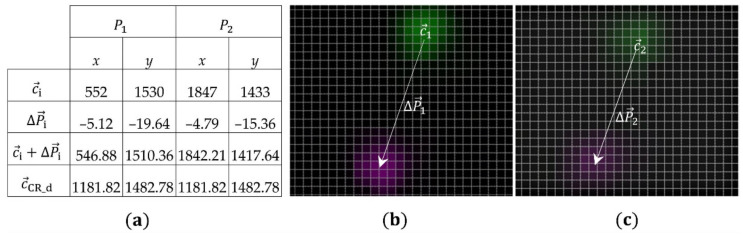
Initial and new positions of points 1 and 2 after multiaxial loading of the structure with ($F_{x}$ + $F_{y}$ + $M_{z}$) and the calculated displacements (**a**–**c**) real displacements of *P*_1_ and *P*_2_ in composite images.

In Figure 10a, the initial positions of points 1 and 2, the calculated displacements by the DIC algorithm, the new positions and the center of rotation, needed for compensation, are shown. In Figure 10b,c, the two images (before and after the load) were composed with each other in Matlab by using the imfuse function to show the real displacements $\Delta P_{1}$ and $\Delta P_{2}$.

With these parameters, the angle of twist φ and the deflections ${\vec{\delta }}_{\mathrm{xy},1}$ and ${\vec{\delta }}_{\mathrm{xy},2}$ can be determined from the calculated displacements $\Delta P_{1}$ and $\Delta P_{2}$. First the angle of twist is calculated. For that purpose, the two vectors ${\vec{v}}_{1}$ and ${\vec{v}}_{2}$ are determined according the Equation (1):

$${\vec{v}}_{1}=\left(\begin{array}{c} P_{2\_}x-P_{1\_}x\\ P_{2\_}y-P_{1\_}y \end{array}\right)=\left(\begin{array}{c} 1295\\ -97 \end{array}\right),{\vec{v}}_{2}=\left(\begin{array}{c} (P_{2\_}x+\Delta P_{2\_}x)-\left(P_{1\_}x+\Delta P_{1\_}x\right)\\ (P_{2\_}y+\Delta P_{2\_}y)-\left(P_{1\_}y+\Delta P_{1\_}y\right) \end{array}\right)=\left(\begin{array}{c} 1295.33\\ -92.72 \end{array}\right)$$

Subsequently, the angle between these vectors (angle of twist φ) is calculated according to Equation (2):

$$\varphi =\arccos\left(\frac{{\vec{v}}_{1}\cdot {\vec{v}}_{2}}{\left|{\vec{v}}_{1}\right|\times \left|{\vec{v}}_{2}\right|}\right)=\arccos\left(\frac{1295\times 1295.33+\left(-97\times -92.72\right)}{\sqrt{1295^{2}+-97^{2}}\times \sqrt{1295.33^{2}+-92.72^{2}}}\right)=0.1894{}^{\circ}$$

Finally, both deflections ${\vec{\delta }}_{\mathrm{xy},1}$ and ${\vec{\delta }}_{\mathrm{xy},2}$ are determined by mean of Equation (3):

$${\vec{\delta }}_{\mathrm{xy},1}=\left(\begin{array}{c} 546.88\\ 1510.36 \end{array}\right)-\left(\begin{array}{c} 1181.82\\ 1482.78 \end{array}\right)-\left(\begin{array}{cc} \cos\left(0.1894\right) & -\sin\left(0.1894\right)\\ \sin\left(0.1894\right) & \cos\left(0.1894\right) \end{array}\right)\left(\left(\begin{array}{c} 552\\ 1530 \end{array}\right)-\left(\begin{array}{c} 1181.82\\ 1482.78 \end{array}\right)\right)\;\;=\left(\begin{array}{c} -634.94\\ 27.58 \end{array}\right)-\left(\begin{array}{cc} \cos\left(0.1894\right) & -\sin\left(0.1894\right)\\ \sin\left(0.1894\right) & \cos\left(0.1894\right) \end{array}\right)\left(\begin{array}{c} -629.82\\ 47.22 \end{array}\right)=\left(\begin{array}{c} -4.967\\ -17.667 \end{array}\right)$$

$${\vec{\delta }}_{\mathrm{xy},2}=\left(\begin{array}{c} 1842.21\\ 1417.64 \end{array}\right)-\left(\begin{array}{c} 1181.82\\ 1482.78 \end{array}\right)-\left(\begin{array}{cc} \cos\left(0.1894\right) & -\sin\left(0.1894\right)\\ \sin\left(0.1894\right) & \cos\left(0.1894\right) \end{array}\right)\left(\left(\begin{array}{c} 1847\\ 1433 \end{array}\right)-\left(\begin{array}{c} 1181.82\\ 1482.78 \end{array}\right)\right)\;\;=\left(\begin{array}{c} 660.39\\ -65.14 \end{array}\right)-\left(\begin{array}{cc} \cos\left(0.1894\right) & -\sin\left(0.1894\right)\\ \sin\left(0.1894\right) & \cos\left(0.1894\right) \end{array}\right)\left(\begin{array}{c} 665.18\\ -49.78 \end{array}\right)=\left(\begin{array}{c} -4.967\\ -17.559 \end{array}\right)$$

As can be seen in the table, the displacements ($\Delta P_{1}$ and $\Delta P_{2}$) of both points were different due to the combined torsional load and the different position of their center of rotation. After determining the angle of twist and compensating for its effect, the two displacements (${\vec{\delta }}_{\mathrm{xy},1}$ and ${\vec{\delta }}_{\mathrm{xy},2}$) became almost identical. Furthermore, the two displacements are averaged and transformed to the coordinate system of the reference sensor, which in our test bench is rotated by 29 degrees to the coordinate system of the image sensor. As the final result we get $\delta_{x}=-12.79\;\mathrm{pixel}$ and $\delta_{x}=-13.09\;\mathrm{pixel}$.

**Figure 11:**
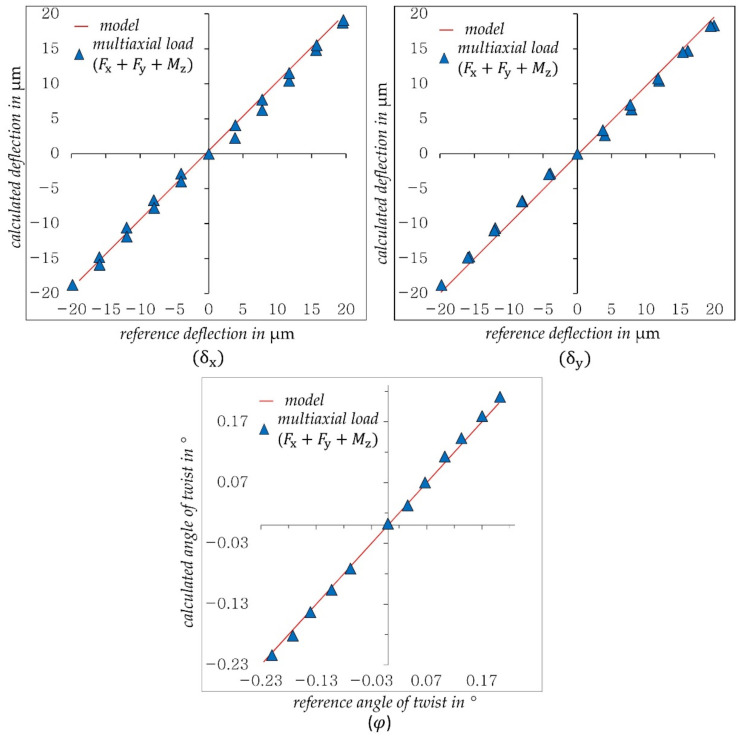
Calculated displacement components ($\delta_{x}$, $\delta_{y}$, φ) when multiaxial loading the structure with ($F_{x}+F_{y}+M_{z}$).

In the same way, the displacement components are determined in the case of combined load $F_{x}+F_{y}+M_{z}$. Figure 11 presents the achieved results for this case.

The calculated deflections $\delta_{x}$, $\delta_{y}$ and the twist angle φ also show good and linear agreement with the reference values. Compared to uniaxial loads in Figure 7, a small deviation for deflection from the reference model can be observed. One possible reason for this deterioration is the screw joints used in the prototype, which are suspected of exhibiting settling behavior under multiaxial loading of the structure.

#### 3.2.5. Results of Multiaxial Loads ($F_{x}+F_{y}+F_{z}+M_{z}$)

**Figure 12:**
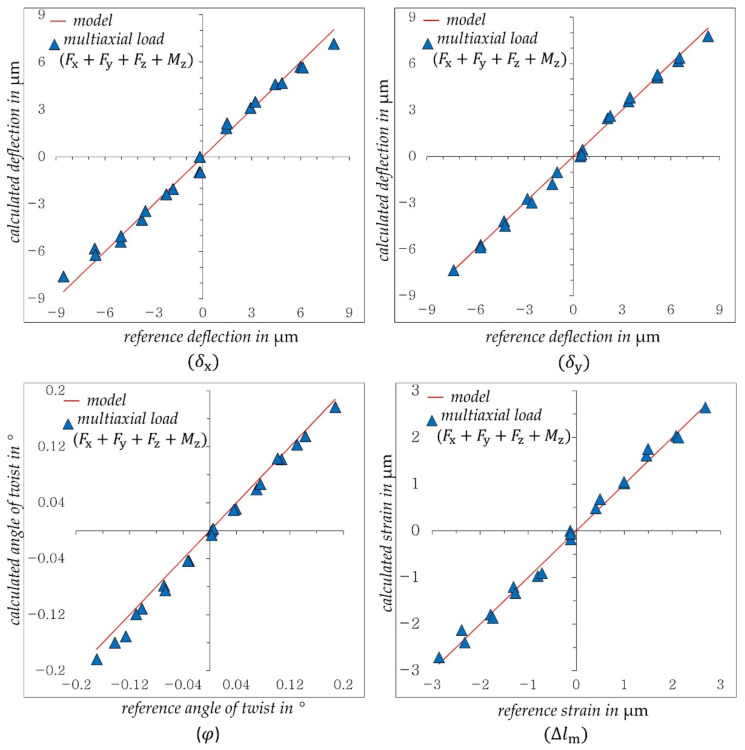
Calculated displacement components ($\delta_{x}$, $\delta_{y}$, φ, Δ$l_{m}$) when multiaxial loading the structure with ($F_{x}+F_{y}+F_{z}+M_{z}$).

In order to avoid any settling behavior in the bolts, the load ranges were reduced in comparison to previous tests as follows: $F_{x\;}$ and $F_{y}$ from ±250 to ±100 N, $F_{z}$ from ±3 to ±1 kN and $M_{z}$ from ±65 to ±50 Nm. After applying the whole load combination ($F_{x}+F_{y}+F_{z}+M_{z}$), the measured displacements $\Delta P_{1-3}$ are calculated to ($\delta_{x}$, $\delta_{y}$, φ and Δ$l_{m}$) according to Equations (1)–(3) and (8). Figure 12 shows the results for this loading case.

The calculated deflections $\delta_{x}$, $\delta_{y}$ and strains Δ$l_{m}$ in Figure 12 show good accuracy and linearity to the reference values despite reduction of the loading range and thus the measuring range. However, the twist angle φ seems to have a higher deviation compared to the previous uniaxial $M_{z}$ und multiaxial ($F_{x}+F_{y}+M_{z}$) load. Despite the relatively high deviation, the displacement components calculated by DIC and the derived equations show very good results. The accuracies achieved in the three loading cases with respect to the used regression models are shown in Table 2. The deviations are specified as the maximum relative error related to the maximum measured value for each displacement component ($\delta_{x}$*,* $\delta_{y}$, φ*,* Δ$l_{m}$).

As can be seen in Table 2, the obtained models have a slight deviation from the theoretical value “1” at uniaxial loads indicating a good robustness of the system in spite of simple manual assembly of the prototype. However, the calculated deviation varies between 2.5% and 10.43%. The displacements ($\delta_{x}$,$\delta_{y}$ Δ$l_{m}$) under uniaxial and whole multiaxial load show the best accuracy and almost the same deviation. The twist angle, on the other hand, shows a higher deviation with multiaxial loading.

#### 3.2.6. Sources of the Measurement Errors

The results shown in Figure 12 were only obtained by tightening the screws and simultaneously reducing the load range. Nevertheless, a deterioration in accuracy, especially compared to the results of uniaxial loading case in Figure 7, remains visible. There was no critical observation regarding the image quality, e.g., disappearance of features under tensile load. Due to the uniqueness of the features in their surroundings, no miscorrelation was suspected by the DIC algorithm. In this particular experiment, the structure length, respectively, the distance between the camera and the object, changed by only ±9 µm, which is within the depth of field for the used imaging system.

However, a major suspicion relates to eccentric axial loading of the structure. Such loading will lead to non-uniform circumferential deformation of the structure and hence tilting of the carrier flanges, which can result in a falsification of the pattern displacement. In this case, the angle of twist would be strongly affected since the pattern displacements under torque loading of the structure are relatively very small compared to the displacements under bending or tensile loading.

**Figure 13:**
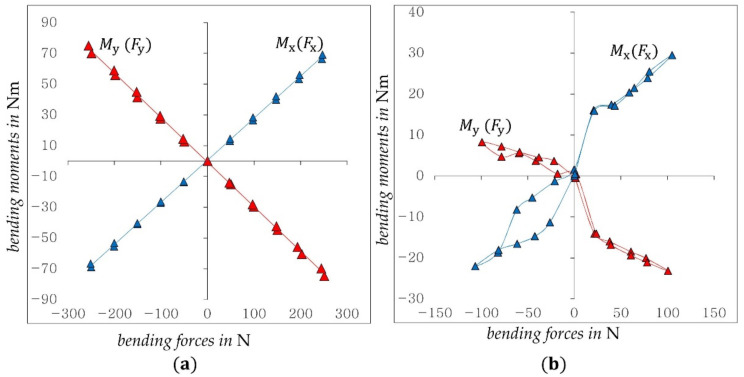
Correlation of bending moments to bending forces in both load cases (**a**) for $F_{x}+F_{y}+M_{z}$ and (**b**) for $F_{x}+F_{y}+F_{z}+M_{z}$.

A look at the bending force and bending moment curves at loading cases ($F_{x}+F_{y}+M_{z}$) and ($F_{x}+F_{y}+F_{z}+M_{z}$) made it clear that in a multiaxial loading combined with axial load, no linear relationship can be found between the bending forces and the bending moments (see Figure 13). This means, that the system behaves differently at each load step.

The clear linearity in load case ($F_{x}+F_{y}+M_{z}$) indicates a homogeneous deformation of the structure during the whole test, this does not seem to be the case in load case ($F_{x}+F_{y}+F_{z}+M_{z}$) and a non-uniform deformation of the structure over the circumference due to eccentric axial loads is assumed.

## 4. Conclusion and Outlook

In the present study we investigated the principal applicability of a camera-based sensor as a structurally integrated multi-axis force/torque sensor within the framework of a prototype. It has been demonstrated that all possible displacements can be detected by the low-cost setup of a camera-based sensor. The use of photomasks with a defined feature position and feature size allows for high-contrast imaging with simple light sources, increasing measurement accuracy and sensitivity with greatly reduced computational effort compared to the conventional application of DIC.

Through simple geometric relationships, both structure deflection and twist angle were clearly observable in the case of multiaxial load, provided the center of rotation was known. For the identification of the center of rotation, a single, preferably torsional, load is required, that results in a pattern rotation around the searched point.

The linear and reproducible curves in all load combinations prove the functionality and simplicity of the presented measurement concept. Despite the achieved high measurement sensitivity of the DIC, resulting from the small and high-contrast features in the designed photomask, deviations of up to 10% were observed. These deviations are partly attributed to eccentric axial loads that cause the structure to deform unevenly around its circumference. This deformation condition causes the carrier plates (flanges) to tilt. Since the introduced sensor concept cannot measure this tilting and take into account its behavior, this load case indicates a key issue for such sensor concepts and must be avoided. In comparison, strain gauge based 6-axes force/torque sensors are usually equipped with separate strain gauges to measure bending moments in addition to bending forces, and therefore do not suffer from this problem in the same way.

In conclusion, however, the presented sensor concept shows several advantages for the field of sensor integrated load-bearing structures and proves to be very attractive for further investigations. The substitution of conventional transducers by contactless measurement enables a multi-axis deformation measurement with higher sensitivity at significantly lower cost. Optical digital measurements have a tremendous advantage in terms of zero-point stability because these structures are usually installed once and referenced with initial installation.

In the future, a suitable process design for sensor integration will be addressed and measurement accuracies will be investigated with respect to the achievable accuracy in the assembly. Once the sensor component has been successfully integrated into the structure without failure-prone screw connections, a comprehensive error analysis of the sensor will be carried out.

## 5. Patents

Patent pending, provisional application number is DE 10 2020 120 192.3.

## Figures and Tables

**Table 1:** Determination of the centers of rotation in both image segments.

	Direct Image Segment				Mirrored Image Segment			
	$P_{1}$		$P_{2}$		$P_{3}$		$P_{4}$	
	*x*	*y*	*x*	*y*	*x*	*y*	*x*	*y*
initial positions ${\vec{c}}_{i}$	552	1530	1847	1433	804	489	1025	1145
linear equations	*y* = −0.0749 *x* + 1571.3				*y* = 2.9683 *x* − 1897.5			
calculated displacement $\Delta {\vec{P}}_{i}$	0.32	−2.12	−1.8	2.2	−1.66	0.55	−5.9	1.97
new position ${\vec{c}}_{i}+\Delta {\vec{P}}_{i}$	554.22	1533.47	1432.59	1852.91	802.34	488.45	1019.1	1146.97
linear equations	*y* = −0.0716 *x* + 1567.4				*y* = 3.0331 *x* − 1944.1			
intersection points	${\vec{c}}_{\mathrm{CR}\_d}=\left(1181.818,\;1482.782\right)$				${\vec{c}}_{\mathrm{CR}\_m}=\left(718.03,233.8\right)$			

**Table 2:** Used models and achieved accuracy of the results in Figure 7, Figure 11 and Figure 12.

Displacement Component	Load Type	Model	Max. Measured Value in µm	Max. Deviation/Max. Measured Value in%
$\delta_{x}$	$F_{x}$	y=1.014 x	20.65	2.58
	$F_{x}+F_{y}+M_{z}$			9.75
	$F_{x}+F_{y}+F_{z}+M_{z}$			2.50
$\delta_{y}$	$F_{y}$	y=1.011 x	20.33	4.22
	$F_{x}+F_{y}+M_{z}$			9.77
	$F_{x}+F_{y}+F_{z}+M_{z}$			4.71
φ	$M_{z}$	y=0.996 x	0.242	2.45
	$F_{x}+F_{y}+M_{z}$			5.53
	$F_{x}+F_{y}+F_{z}+M_{z}$			10.43
Δ$l_{m}$	$F_{z}$	y=1.0215 x	8.22	2.66
	$F_{x}+F_{y}+F_{z}+M_{z}$			3.18

## Data Availability

Not applicable.
